# T2 hypointense lesions in the parapharyngeal space: a diagnostic challenge

**DOI:** 10.1007/s11547-025-02149-x

**Published:** 2025-11-13

**Authors:** Edith Vassallo, Emma Tabone, Reuben Grech, Marco Ravanelli, Ivan Zorza, Valerio Mazza, Giulia Petrilli, Lorenzo Ugga, Davide Farina, Roberto Maroldi, Minerva Becker

**Affiliations:** 1 Medical Imaging Department, Mater Dei Hospital, Imsida, MSD2090 Malta; 2 University Hospital of Wales, Cardiff and Vale Health Board, Cardiff, UK; 3 Department of Radiology, University of Brescia, P.Zzale Spedali Civili 1, 25123 Brescia, Italy; 4 Santa Chiara Hospital, Largo Medaglie d’oro, 9, 38122 Trento, Italy; 5 Department of Pathology, University of Brescia, P.Zzale Spedali Civili 1, 25123 Brescia, Italy; 6 Department of Advanced Medical and Surgical Sciences, University of Campania “Luigi Vanvitelli”, Piazza Miraglia 2, 80138 Naples, Italy; 7 Division of Radiology, Diagnostic Department, Geneva University Hospitals, Genève, Switzerland

**Keywords:** Parapharyngeal space, T2 hypointense lesions, Melanoma, Granulomatous lesions, Fibrous lesions

## Abstract

The parapharyngeal space is a complex anatomical site in the head and neck which may harbour clinically occult pathology given its deep-seated location. The vast majority of parapharyngeal space lesions are of intermediate or hyperintense signal on T2W sequences. This review focuses on T2 hypointense parapharyngeal space lesions which are rare and may constitute a diagnostic dilemma. We present the differential diagnosis of these lesions, highlighting the pertinent radiological findings and identifying a histological correlation for the low T2 signal. A brief discussion of the physics principles accounting for these imaging features is also included. We propose a diagnostic algorithm to facilitate diagnosis and avoid unnecessary biopsy, whenever possible.

## Introduction

**Fig. 1:**
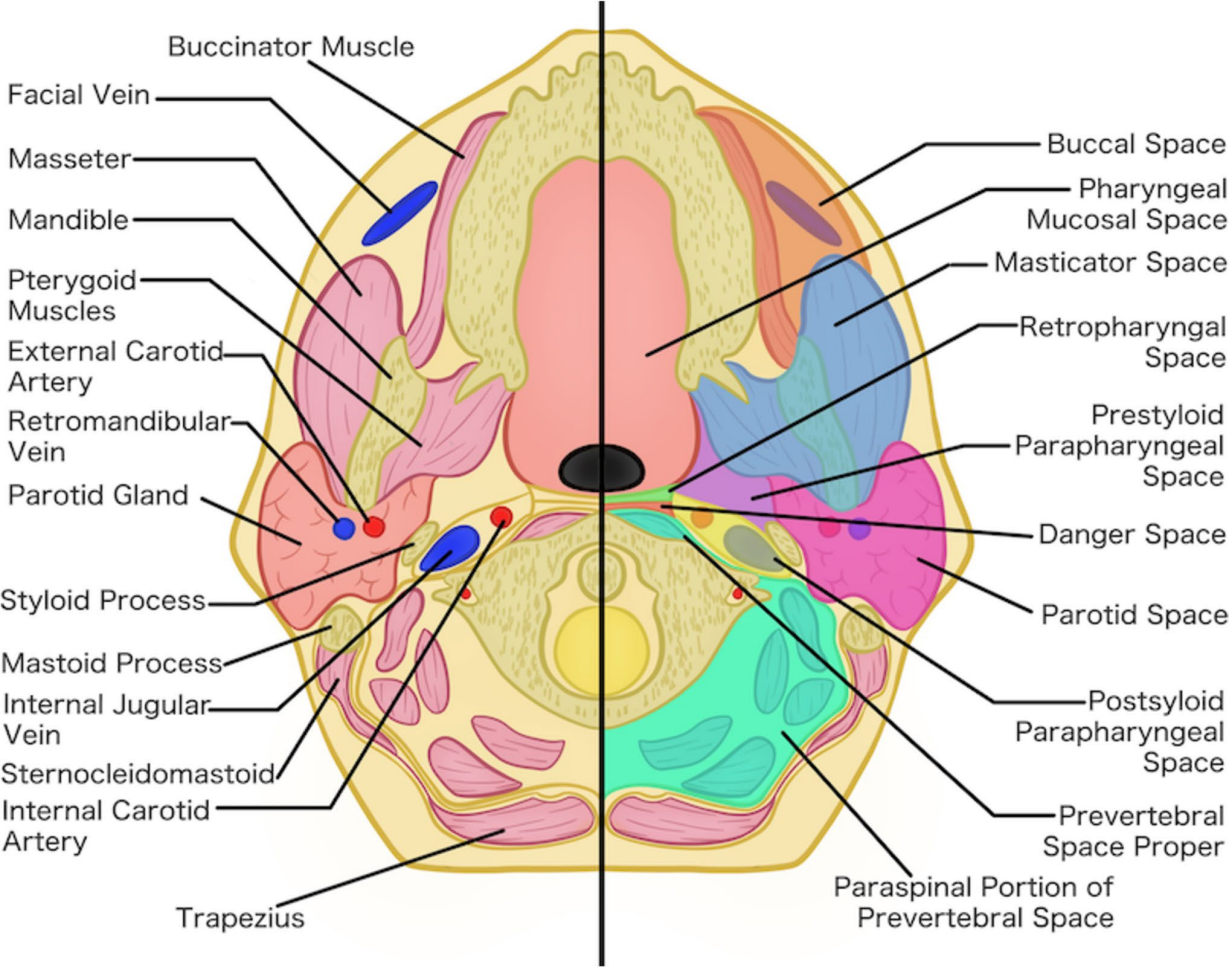
Schematic drawing of the parapharyngeal space demonstrating the pre- and post-styloid compartments and its relationship to the other neck spaces

The parapharyngeal space (PPS) is a paired, fat-filled, lateral suprahyoid neck space extending from the skull base down to the level of the hyoid bone. It lies medially to the masticator and parotid spaces, laterally to the pharyngeal mucosal space and anterolaterally to the retropharyngeal space (Fig. 1). It is divided into pre- and post-styloid compartments by the tensor-vascular styloid fascia. The prestyloid compartment contains predominantly fat, few minor or ectopic salivary glands, the internal maxillary artery, ascending pharyngeal artery, pterygoid venous plexus, and a small branch of the mandibular division of the trigeminal nerve, supplying the tensor veli palatini muscle. The post-styloid compartment houses the internal carotid artery, internal jugular vein, 9th to 12th cranial nerves, glomus bodies, and the cervical sympathetic chain [1, 2].

PPS masses include tumours and tumour-like lesions. These are uncommon and account for less than 1% of all head and neck masses [1].

Most tumours of the PPS arise from adjacent spaces. True PPS tumours are rare, with 80% of them being benign, predominantly represented by pleomorphic adenomas, arising either from the deep lobe of the parotid gland or in ectopic salivary glands [3]. Tumour-like lesions involving the PPS may include branchial cleft cysts, vascular malformations, and granulomatous diseases [4, 5].

In the extracranial head and neck, the signal intensity of tumours and tumour-like lesions on T2W images provides important diagnostic information, as benign lesions typically have a high signal intensity on T2W sequences whereas malignant lesions, in particular carcinomas, often have a characteristic intermediate signal intensity. There are, however, a number of exceptions. For instance, chondrosarcoma is distinctly bright on T2 sequences, whereas conversely, pleomorphic adenomas may sometimes return intermediate signal on T2 and even restrict diffusion. Hence, signal intensity alone is often not enough to distinguish benign from malignant lesions, and other morphological features such as size, depth, and signal heterogeneity on T2 can help to improve the ability of MRI to distinguish benign from malignant lesions [6].

In contrast, hypointense lesions on T2W images are uncommon and they often constitute a diagnostic challenge [7]. Their differential diagnosis is very broad, and several theories have been put forward to understand the significance of this specific appearance. Given its deep-seated location, biopsy or surgical exploration of the PPS is not straightforward; therefore, a precise diagnosis, avoiding unnecessary invasive procedures, is of utmost importance. Nevertheless, to the best of our knowledge, the existing literature on T2 hypointense lesions of the PPS is virtually non-existent. There is thus no dedicated paper or article focussing specifically on the diagnostic algorithm of T2 hypointense PPS lesions. This educational review aims to fill this gap.

We attempt to describe the histological features and physics principles that account for such distinct signal characteristics. Further aims of this review include providing a diagnostic algorithm to guide radiologists when dealing with these entities, using a multiparametric and multimodality approach.

## Physics of T2 hypointensity

T2W spin echo images are basic MRI sequences which depend on two important parameters: long repetition time (TR) > 2000 ms and long time to echo (TE) > 80 ms [8]. The T2 value is tissue specific. Substances with a large portion of free water appear bright on T2W images because of a longer T2 relaxation time. Conversely, substances which return low signal on T2W sequences have a short T2 relaxation time. Lesions that determine hypointensity on T2W sequences can be arbitrarily divided into two main groups, namely an “inanimate” group (which includes lesions containing metallic compounds, certain minerals, and artefactual phenomena *like flow voids*), and an “animate” group (including hypocellularity, acellularity, abundant collagen deposition [9, 10], highly proteinaceous content, and fibrous tissue [11, 12].

Paramagnetic substances include blood products (deoxyhemoglobin, methaemoglobin, and haemosiderin), melanin, ferritin, magnesium, and calcium. Paramagnetic substances shorten T2 relaxation times because their unpaired electrons induce local magnetic field inhomogeneities, which -in turn- increase dephasing [13].

Flow voids occur on T2W sequences because moving blood exits the sampled area faster than TE/2, and their long TE makes these flow voids more conspicuous than on sequences with shorter TE [8].

Within a solution, the concentration of macromolecular proteins will determine the T1 and T2 relaxation properties. As the protein content of the solution increases, the T2 relaxation time decreases [11].

Likewise, tumours with abundant collagen, low cellularity and high nucleus-to-cytoplasm ratios limit molecular motion, induce local magnetic field inhomogeneities, which contribute to increased dephasing and, therefore, T2 shortening. According to M Sundaram et al. it is the histological composition of the tumour which affects the signal intensity on T2W sequences and not the histological diagnosis [9].

Finally, in the absence of mobile protons or in the presence of very few mobile protons, e.g. air or cortical bone, a low signal intensity is observed on all pulse sequences, including T2W imaging [14].

## Diagnostic approach to T2 hypointense lesions

When discussing T2 hypointensity, the authors refer to signal which is visually comparable to or more hypointense than the T2 signal of normal muscle. In view of the diverse causes of T2 hypointensity, the differential diagnosis of T2 hypointense lesions in the PPS can be narrowed down by assessing the signal intensity of the lesion on other MR pulse sequences, including contrast-enhanced sequences, and diffusion-weighted imaging (DWI). This multiparametric approach, occasionally combined with CT, as well as the location of the lesion within the PPS, and its pattern of deep spread are crucial for the correct diagnosis [15].

A lesion which is of low signal on T2W sequences may return low or high signal on T1. If it is hyperintense on T1, then a melanotic melanoma should be considered and the necessary diagnostic work-up undertaken. If it is hypointense on T1, intravenous Gadolinium administration should be the next step in the patient’s work-up. Tissue enhancement can help distinguish tumours (e.g. paragangliomas) and inflammatory conditions (e.g. myofibroblastic tumour and granulomatous conditions) from highly proteinaceous lesions which do not enhance (e.g. sinonasal mucoceles).

## Lesions containing calcium

### Epidemiology and relevance in the PPS

Calcium-containing lesions in the PPS are exceedingly rare. Reported entities include mesenchymal chondrosarcoma (with only four cases documented in the literature) [16], extensively ossified pleomorphic adenoma, calcified metastatic thyroid carcinoma [17], and ectopic parapharyngeal meningioma [18]. The literature is limited to a very few case reports of these lesions. Calcified retropharyngeal lymphadenopathy extending into the PPS may occur in neuroblastoma and should raise suspicion for this diagnosis particularly in children.

Imaging features and histopathological correlation.

On MRI, calcium typically returns low signal on T2W sequences. This is related to the mineral content of calcium and other calcified matrix, which limits the presence of mobile protons and produces susceptibility effects that shorten T2 relaxation times. CT is particularly useful in confirming calcification and narrowing the differential diagnosis.

Key differentiating features:

- The extreme rarity of calcified PPS tumours such as mesenchymal chondrosarcoma or ossified pleomorphic adenoma makes radiological–pathological correlation essential.
- Calcified metastatic thyroid carcinoma should be suspected when a calcified PPS lesion is present with a relevant clinical history [17].
- Calcified retropharyngeal lymphadenopathy with PPS extension should raise suspicion for neuroblastoma, especially in children.

## Lesions containing melanin

### *Melanoma (*Fig. 2*)*

**Fig. 2:**
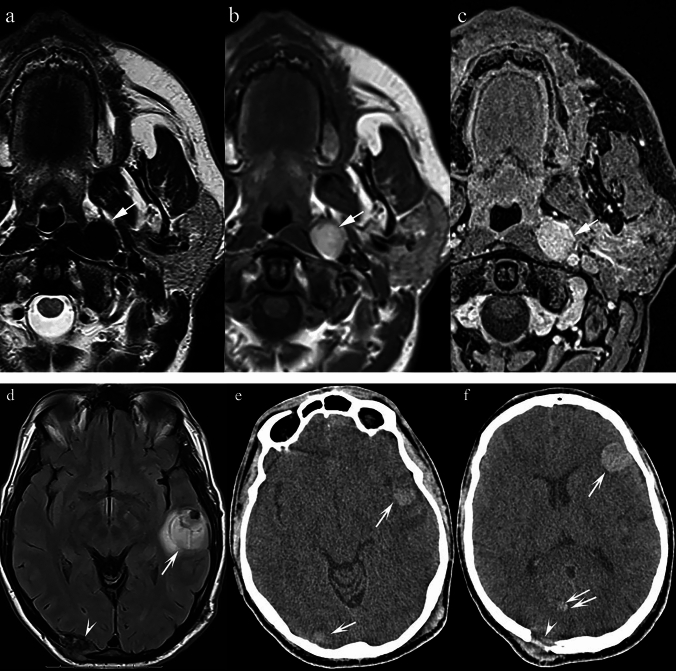
Patient with a history of lower lip melanoma for which he underwent local excision accompanied by radical bilateral neck dissection. There was no history of thyroid malignancy. a Axial T2 TSE at the level of the first cervical vertebra reveals a modestly sized, well-demarcated, low signal intensity mass in the left parapharyngeal space which is clearly separate from the tail of the parotid gland (arrow). b Axial T1 SE sequence without contrast at the same level reveals the lesion to be of very high signal intensity relative to the parotid gland (arrow). c T1 VIBE post-contrast demonstrates homogenous enhancement (arrow). d FLAIR sequence of the brain demonstrates a large haemorrhagic metastasis in the left temporal lobe (arrow) and a cortical breach in the occipital bone on the right side due to another metastatic deposit (arrowhead) e Non-contrast CT scan at the level of the midbrain, demonstrates a haemorrhagic metastasis in the left temporal lobe (single arrow) and a metastasis in the right occipital lobe (double arrow). f Non-contrast CT scan at the level of the thalamus, shows a haemorrhagic metastasis in the left frontal lobe (single arrow), in the right occipital lobe (double arrow) and a bone metastasis in the occipital bone on the right side (arrowhead)

#### Epidemiology and relevance in the PPS

To date, melanoma metastases to the PPS have not been documented in the literature. The presented case (Fig. 2) was not histologically confirmed, but the absence of thyroid malignancy — the main differential diagnosis — supported the presumptive diagnosis. For context, only 112 cases of PPS metastases from thyroglobulin-containing thyroid lesions have been reported [19].

Imaging features and histopathological correlation:

Melanotic melanomas are characteristically bright on T1 sequences due to the paramagnetic effect of melanin [20], and hypointense on T2W sequences. Melanoma metastases can demonstrate variable MR patterns depending on whether they are melanotic or amelanotic. In addition, intralesional haemorrhage is common, producing further T1 and T2 shortening due to the presence of methaemoglobin [27].

The signal properties of melanoma are related to melanin, which contains free radicals [21] [22, 23] and has high affinity for metal ions such as iron, copper, manganese, and zinc [24] [25, 26]. These properties shorten both T1 and T2 relaxation times. Haemorrhagic change within metastases further accentuates hypointensity by introducing blood breakdown products with paramagnetic effects [27].

Key differentiating features:

- T1 hyperintensity in a PPS lesion should strongly suggest melanocytic melanoma.
- Differentiating melanoma from thyroid metastases is crucial; thyroid origin is more common but typically confirmed with thyroglobulin markers and a pertinent history (19).

## Lesions with turbulent or rapid flow

### *Paraganglioma (*Figs. 3, 4*)*

**Fig. 3:**
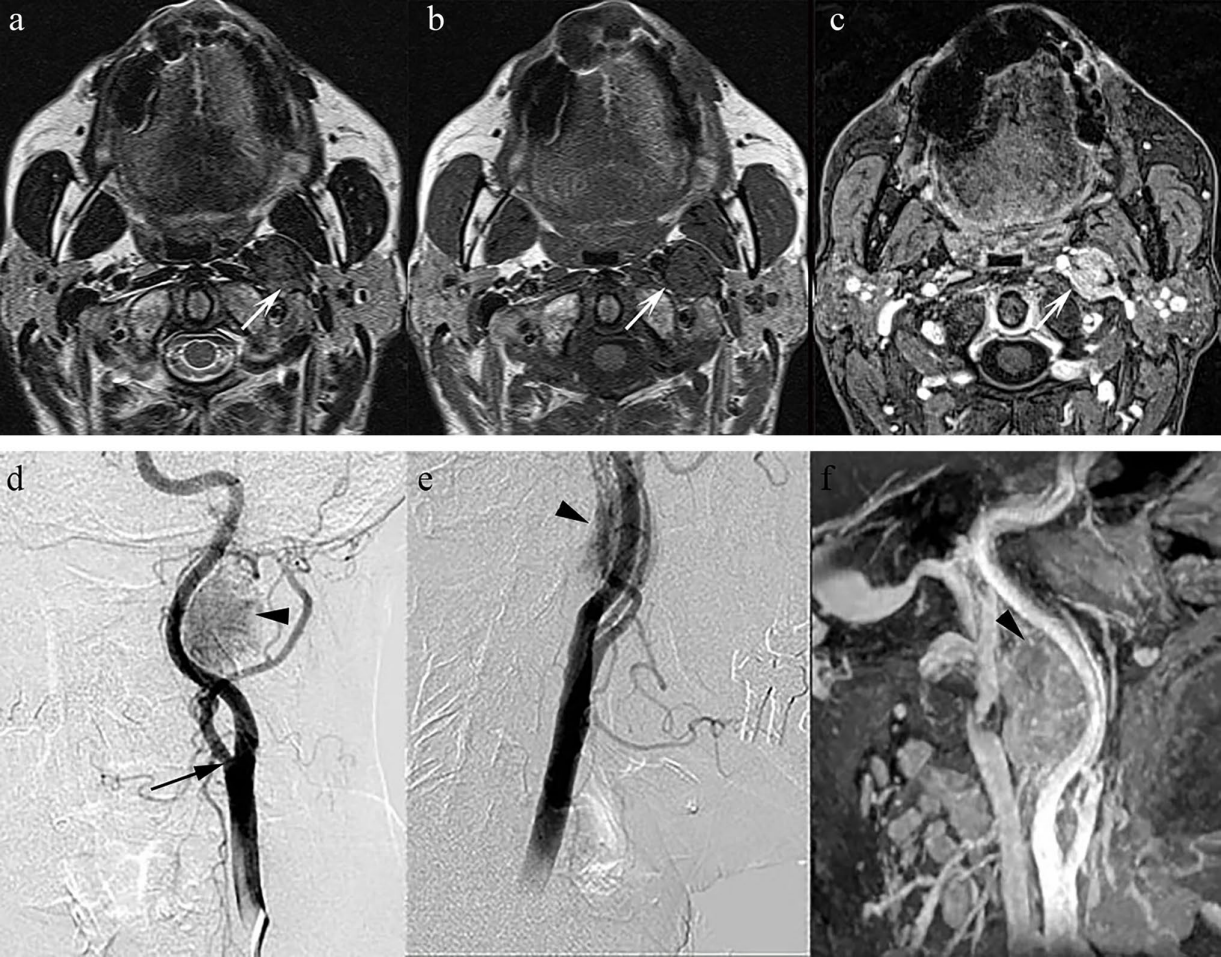
a Axial T2 TSE sequence at the level of the C1 vertebra shows a modestly sized, round T2 hypointense lesion in the left parapharyngeal space (arrow). b Axial T1 SE sequence at the same level indicates that the lesion remains hypointense (arrow). c Axial T1 VIBE post-contrast demonstrates avid enhancement (arrow); the internal carotid artery is displaced anteromedially and the internal jugular vein posterolaterally. (d, e) AP and oblique views from a digital subtraction angiographic series: the lesion does not cause splaying at the bifurcation of the common carotid artery (arrow) excluding a carotid body tumour. f Reconstruction from the T1 VIBE post-contrast sequence confirms the findings of the DSA making it highly suggestive of a vagal paraganglioma

**Fig. 4 (:**
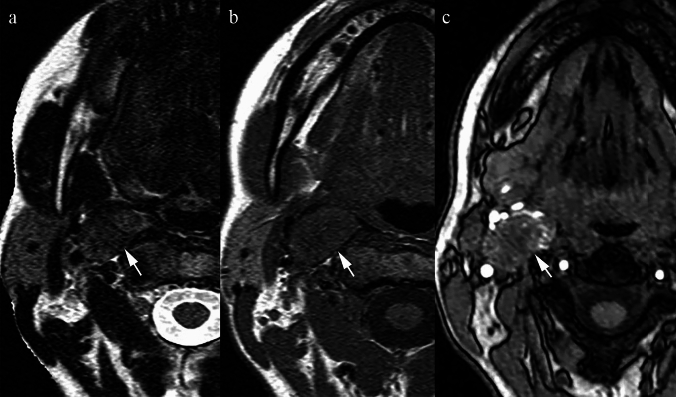
a) Axial T2 TSE sequence depicts an oval-shaped low signal intensity mass in the right parapharyngeal space (arrow). It maintains its low signal intensity on T1 (b) (arrow) and shows only modest enhancement on the post-contrast T1 VIBE sequence (c) displacing the carotid vessels anterolaterally and the internal jugular vein in a posterolateral direction. This was a histologically proven paraganglioma of the cervical sympathetic chain

#### Epidemiology and relevance in the PPS

Head and neck paragangliomas are rare neural crest-derived tumours. They are highly vascular and usually benign. Common sites include the carotid bifurcation, jugular bulb, tympanic plexus, and vagal nerve. In the PPS, vagal paragangliomas, carotid body tumours, and sympathetic chain paragangliomas may occur [28].

Imaging features and histopathological correlation.

Paragangliomas in the PPS may appear hypointense, intermediate, or even hyperintense on T2 sequences [28]. They typically show avid post-contrast enhancement and may demonstrate flow voids due to their vascularity. Vessel displacement patterns (e.g. anteromedial displacement of the carotid artery and posterolateral displacement of the jugular vein) can help localise the tumour’s origin.

Histologically, paragangliomas display a characteristic “zellballen” (nesting/trabecular) cellular arrangement within a rich vascular network [29]. Abnormal vascular morphology, stromal abundance, melanin-like pigment, and occasional osseous metaplasia all contribute to variable T2 hypointensity.

Key differentiating features:

- Avid, homogeneous contrast enhancement and flow voids on T2W sequences are hallmark features.
- Displacement patterns of the carotid artery and jugular vein help distinguish between vagal, carotid body, and sympathetic chain paragangliomas.
- Unlike schwannomas, paragangliomas tend to show avid enhancement and vascular flow-related features characteristically referred to as a “salt and pepper” sign, whereas schwannomas as more likely to be associated with central cystic change or a “target sign” [28].

## Highly cellular lesions

### *Undifferentiated squamous cell carcinoma metastasis (*Fig. 5*)*

**Fig. 5:**
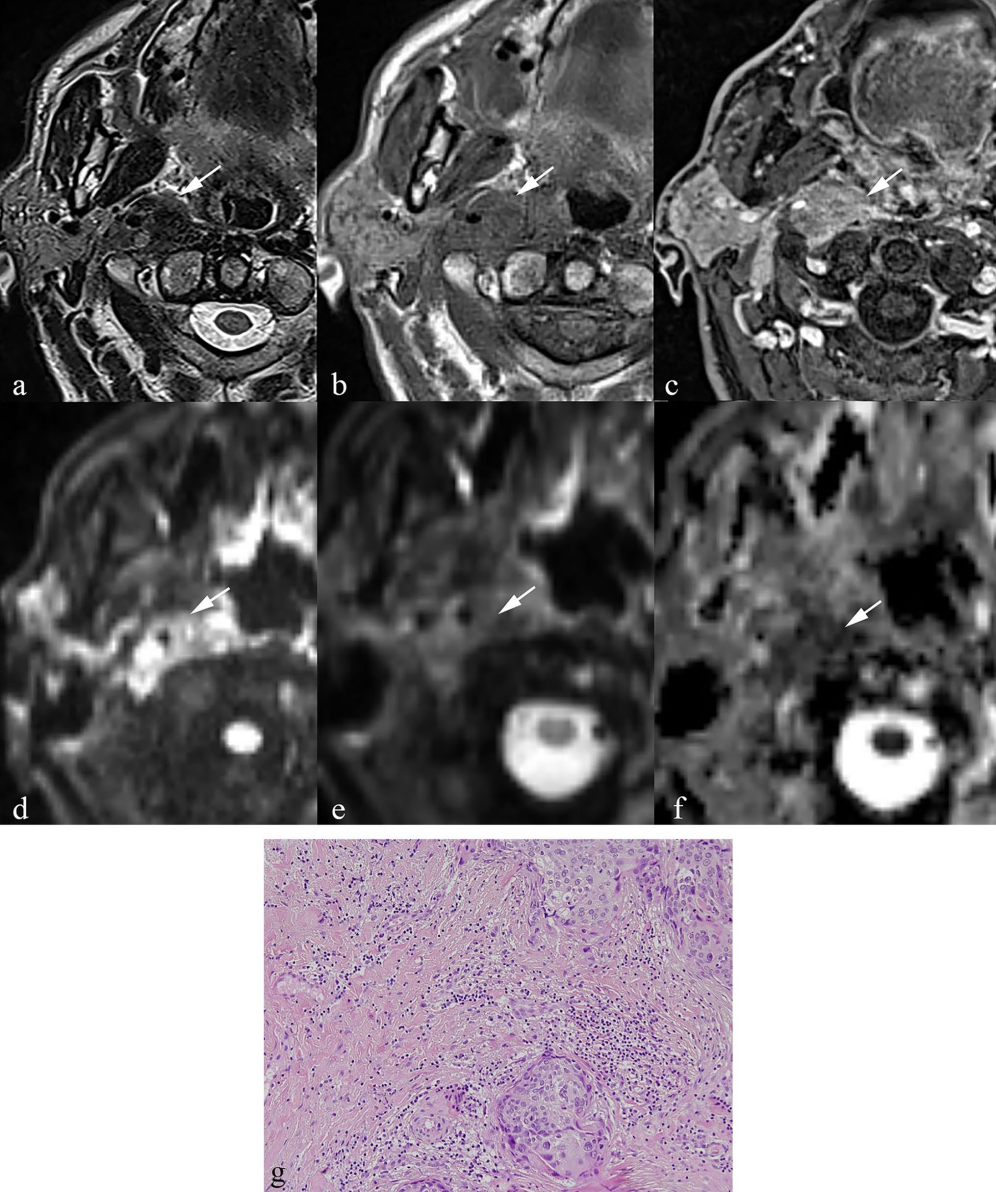
(a) Axial T2 TSE at the level of the parotid body demonstrates a hypointense mass in the right parapharyngeal space completely encasing the carotid space vessels (arrow) with low to intermediate signal intensity on the T1 SE sequence (b) and modest, homogeneous contrast enhancement on the T1 VIBE (c). The lesion exhibits strong restriction with no significant elevation of signal in the B0 (arrow) (d), significantly raised signal in B1000 (arrow) (e), and very low signal on the ADC Map (f) (arrow) in keeping with a malignant tumour (g) Histology specimen shows fragments of lymphoid tissue at the site of cancer proliferation of aetiology referable to undifferentiated squamous cell, organized in solid comedic clusters, in the presence of voluminous elements of bizarre aspect, with diffuse immunoreactivity for p16 and p63. There is focal epithelial lining of the respiratory/transitional type associated with lymphoid populations. The lesion is in keeping with an undifferentiated squamous cell carcinoma metastasis likely secondary to an oropharyngeal primary

#### Epidemiology and relevance in the PPS

PPS nodal metastases most often arise from primaries in the pharynx or sinonasal tract. Rarely, they may originate from papillary thyroid carcinoma, with only 10 such cases reported [30]. In the authors’ experience, undifferentiated squamous cell carcinoma metastasis to the PPS is extremely uncommon (see Fig. 5).

Imaging features and histopathological correlation.

On MRI, these metastases appear T2 hypointense or low-intermediate in signal and often show modest, homogeneous contrast enhancement. They typically demonstrate restricted diffusion with low ADC values, consistent with malignancy. Encasing or invading carotid space vessels may be observed, reflecting aggressive behaviour.

The hypointense T2 signal is due to a desmoplastic reaction, whereby stromal fibroblasts proliferate and lay down connective tissue in response to tumour invasion [31]. This fibrotic tissue acts as a pseudo-capsule around the metastasis, increases collagen content, and therefore shortens T2 relaxation times.

Key differentiating features.

- Diffusion restriction with low ADC values distinguishes metastases from benign PPS lesions.
- Desmoplastic reaction and extracapsular spread are key histological features, not seen in primary benign tumours.
- Clinical history of an oropharyngeal or sinonasal primary strongly supports the diagnosis.

### *Warthin Tumour (*Fig. 6*)*

**Fig. 6:**
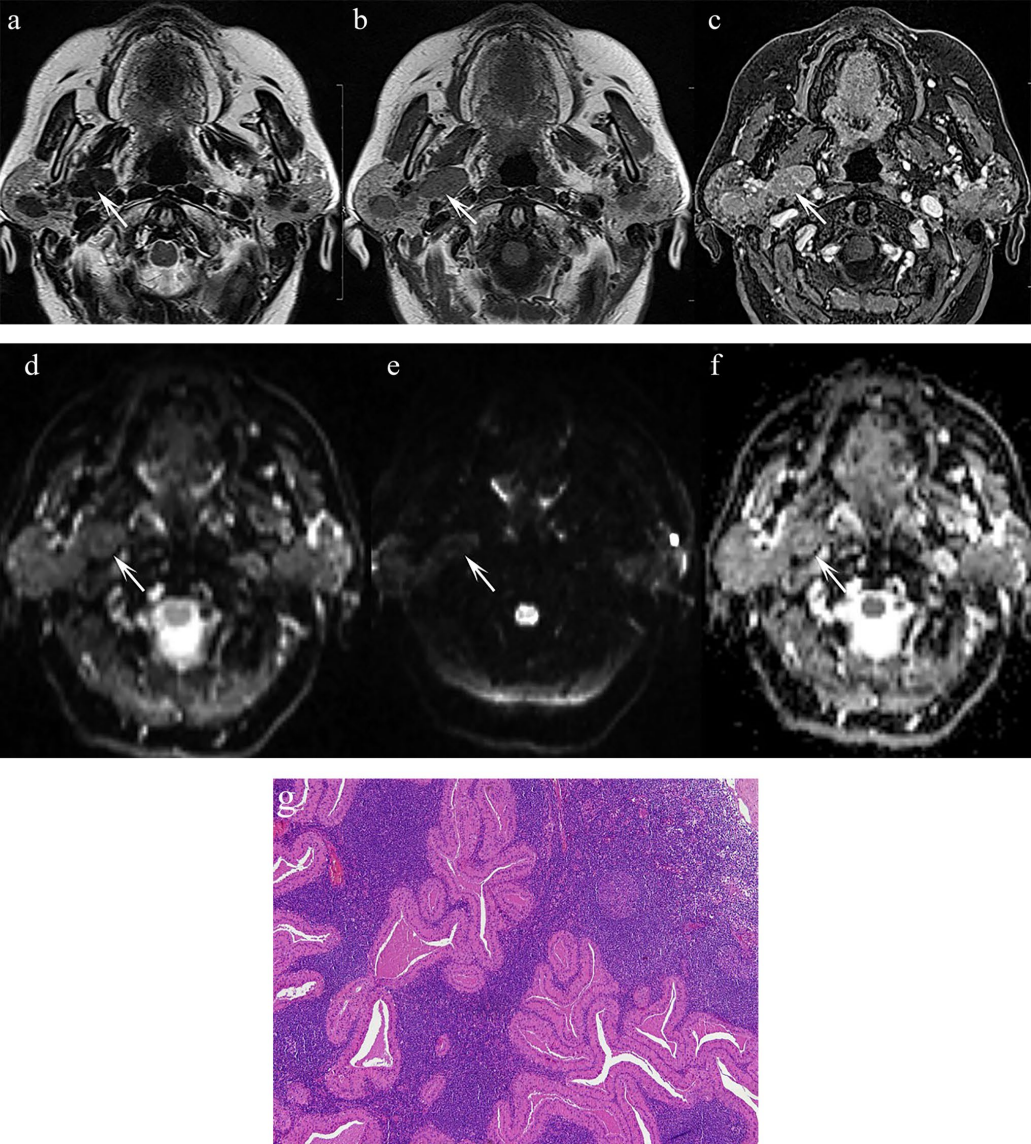
a Axial T2 TSE at the level of the parotid body, demonstrates bilateral variably sized, hypointense lesions in both parotid glands, largest on the right protruding into the parapharyngeal space (arrow). It has no identifiable fat plane separating it from the deep lobe of the parotid gland. b Axial T1 SE at the same level reveals the lesions to be of intermediate signal intensity. c T1 VIBE axial sequence at the same level: the lesions demonstrate mild enhancement with respect to the parotid gland though the pattern of enhancement is homogeneous. d DWI B0 image in the axial plane shows that the lesions have signal characteristics analogous to that of the parotid gland, confirmed on the axial DWI B1000 e and the ADC Map f. g Histology slide demonstrating epithelial cell proliferation associated with a lymphoid stroma, referable to a Warthin tumour, with alternating areas of architecture that are solid and cystic. The tumour is at times limited by a fibrous pseudo-capsule and at times demonstrates a growth as if from a pre-exiting lymph node structure. Both lesions were histologically proven to represent Warthin tumours

#### Epidemiology and relevance in the PPS

Warthin tumour is the second most common benign parotid neoplasm after pleomorphic adenoma. It typically arises in the parotid gland but very rarely involves the minor salivary glands [33]. Aside from the case we present in this article, and up until 2006, there were only two other cases documented in the English literature of Warthin tumour arising in the deep lobe of the parotid gland extending into the PPS [34]. With respect to such lesions being situated in the PPS, interestingly, until 1997 only one case had been reported in the literature. This originated in the deep portion of the parotid gland with extension into the PPS [35].

Imaging features and histopathological correlation.

On MRI, Warthin tumours often appear hypointense on T2 sequences [32]. They show intermediate T1 signal and mild, usually homogeneous, post-contrast enhancement. Diffusion characteristics are generally similar to the parotid gland itself.

These tumours are composed of an epithelial component with a lymphoid stroma. T2 hypointensity is attributed to fibrosis, haemosiderin deposition from prior haemorrhage, and the dense lymphoid and epithelial elements [33].

Key differentiating features.

- Unlike pleomorphic adenomas, which are usually hyperintense to intermediate signal on T2, Warthin tumours classically return low T2 signal.
- Patient demographics (often older males, sometimes with smoking history) can also aid differentiation.

### *Inflammatory myofibroblastic tumour (IMT) and Inflammatory pseudotumour (IPT) (*Figs. 7, 8, 9*)*

**Fig. 7:**
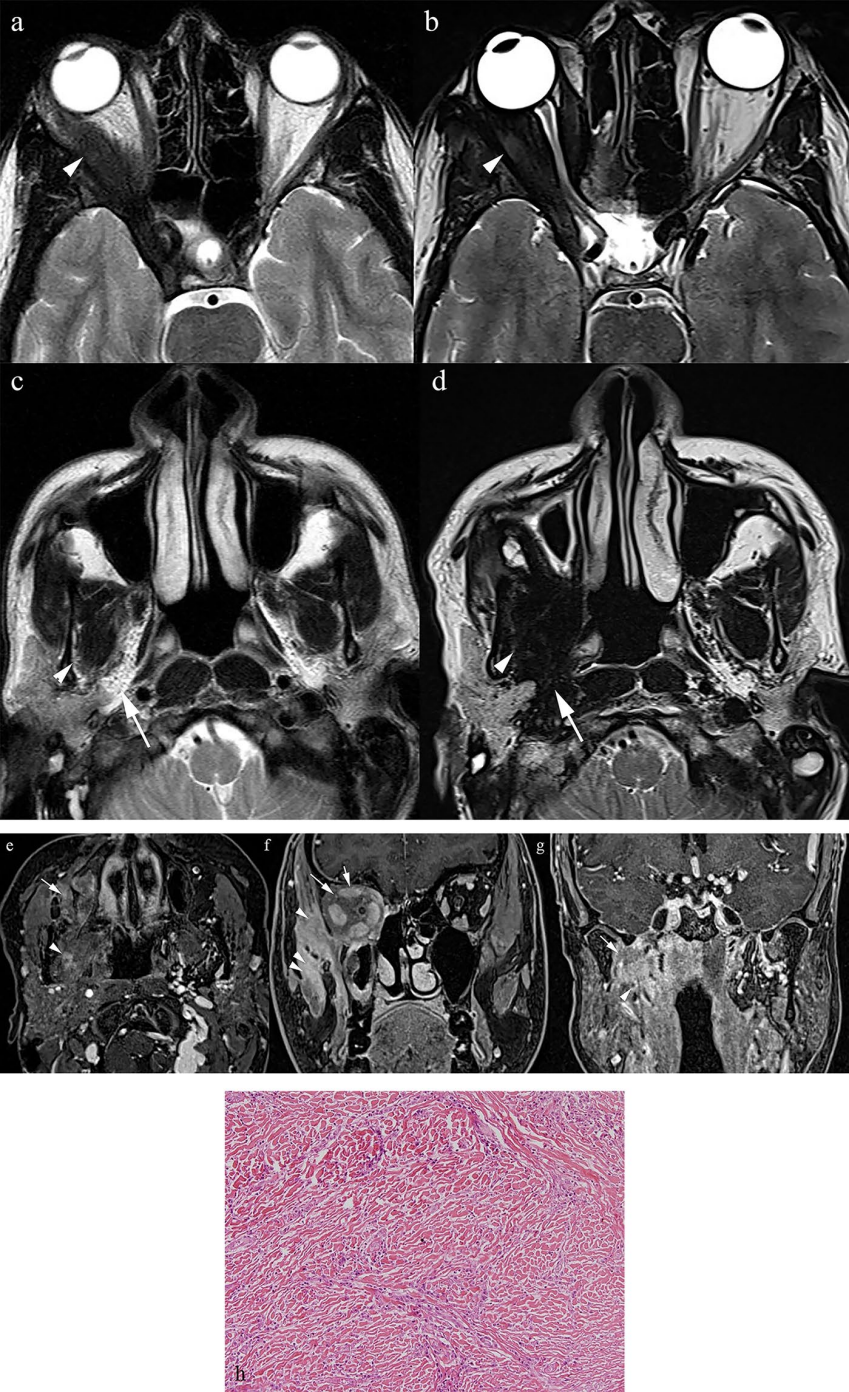
a Axial T2 TSE at the level of the ocular globes in 2005: infiltration of the lateral aspect (< 50%) of the right intraconal fat (arrowhead) with T2 hypointense tissue. b Axial T2 TSE same level in 2015: marked disease progression now involving the entire intraconal fat (arrowhead), returning profoundly low T2 signal. c Axial T2 TSE at the level of the tensor and levator veli palatini taken in 2005. The latter and the parapharyngeal space are clearly visible bilaterally. d Axial T2 at the same level in 2015: there is now complete infiltration of the masticator (arrowhead) and parapharyngeal spaces by disease. e Axial T1 VIBE post-contrast: heterogenous contrast enhancement in the retromaxillozygomatic space on the right (arrow) and ipsilateral pterygoid muscles (arrowhead). f Coronal reconstructed T1 VIBE post-contrast at the level of the orbits posteriorly: enhancement and thickening of the intraconal fat (arrow) and extrinsic ocular muscles and avid enhancement of the temporalis and masseter muscles (arrowheads). g Coronal VIBE sequence at the level of Meckel’s cave: avid enhancement of the right parapharyngeal space (arrow). 3 h Histology specimen shows proliferation of fusiform elements, associated with a rich inflammatory component, prevalently lymphoplasmacellular, also comprising the formation of follicles. The lesion alternates between areas of high cellularity with an appearance that is fibroinflammatory characterised by numerous histiocytes and foamy cytoplasm and areas that are hypocellular characterised by the presence of stroma with abundant scar-like collagen. This was a confirmed inflammatory myofibroblastic tumour

**Fig. 8:**
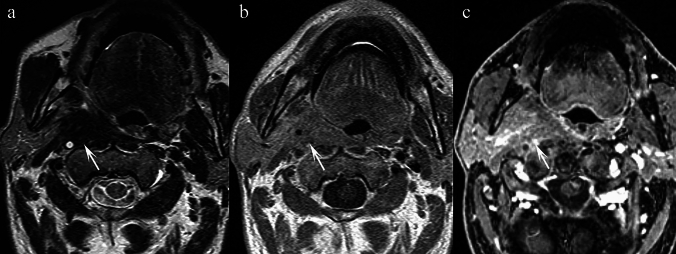
A separate case of histologically confirmed inflammatory myofibroblastic tumour a T2 TSE sequence at the level of the second cervical vertebra, demonstrates an ill-defined infiltrative mass in the right parapharyngeal space with signal intensity analogous to that of muscle (arrow). It is seen to encase the internal carotid artery which displays abnormal high signal. b T1 SE sequence at the same level: the lesion displays intermediate signal intensity (arrow). c T1 VIBE post-contrast: enhancement of the lesion is sparse (arrow). No contrast filling is seen within the internal carotid artery suspicious for intraluminal thrombus

**Fig. 9:**
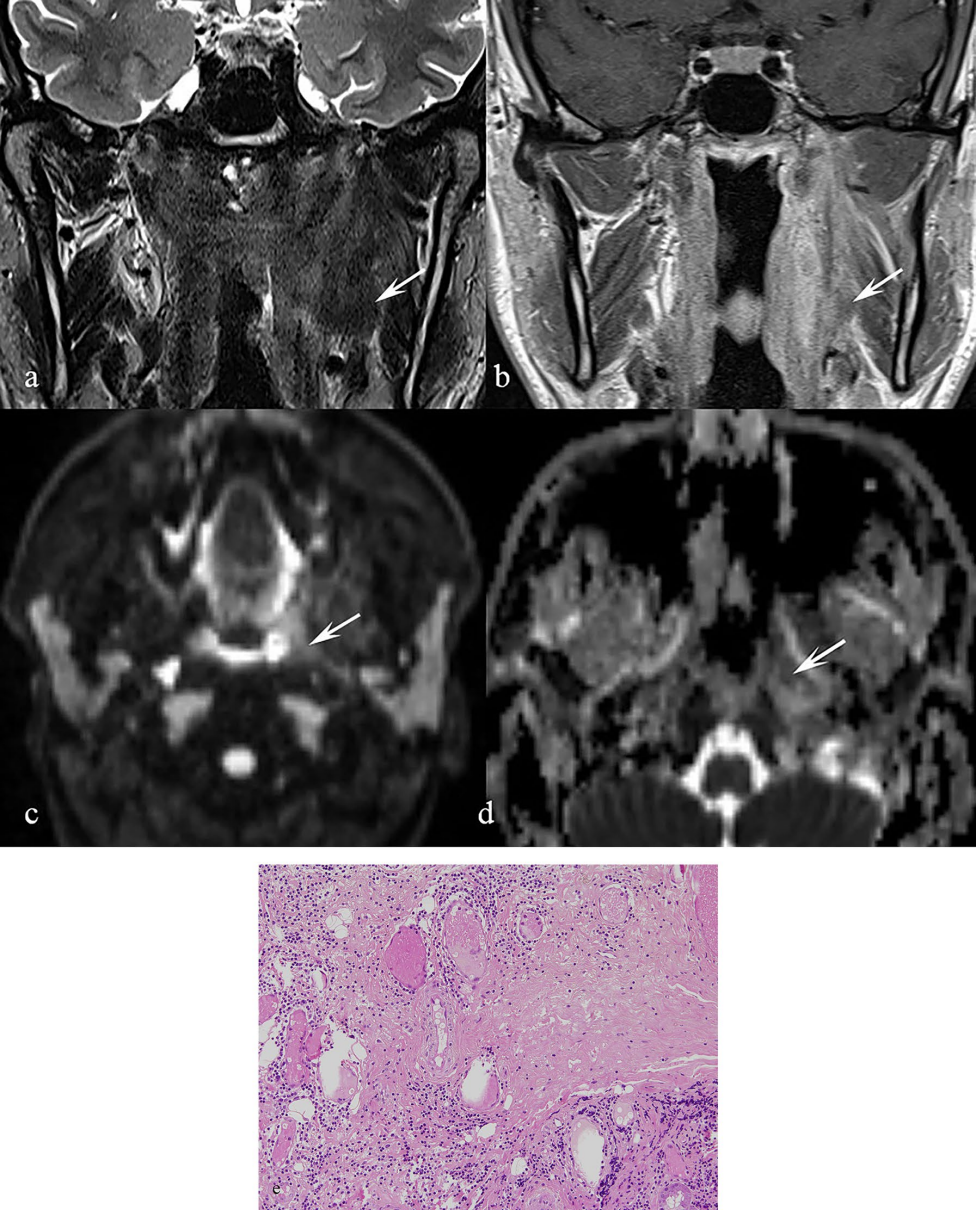
a Coronal T2 TSE demonstrates an infiltrative, low signal intensity mass (arrow) completely obliterating the left parapharyngeal space. b Coronal post-contrast T1 SE sequence shows homogeneous enhancement of the left parapharyngeal space (arrow) and modest enhancement of the medial margins of both pterygoid muscles (arrow). c Axial DWI B1000 at the level of the parotid bodies demonstrates high signal (double arrow) that corresponds to low signal on the ADC map d in keeping with restricted diffusion (arrow). e Image from the histology specimen shows a fibroinflammatory process that contains a population of polymorphic lymphocytes with a rich component of macrocytic lymphocytes, without evidence of granulomas. There are granulocytic components including eosinophils with occasional formation of microabscesses. This was confirmed to represent an inflammatory pseudotumour

#### Epidemiology and relevance in the PPS

IMT and IPT are rare, idiopathic lesions that can involve the PPS. They are uncommon in the maxillofacial region and often mistaken for malignant tumours due to their clinical and radiological appearance [7, 36]. Reported sites include the oral cavity, submandibular gland, larynx, epiglottis, maxillary sinus, and PPS [37–39].

Imaging features and histopathological correlation.

On MRI, IMTs and IPTs typically appear as T2 hypointense or muscle isointense infiltrative masses. They may encase vessels, show variable post-contrast enhancement (often modest or heterogeneous), and demonstrate restricted diffusion. Their imaging features often overlap with malignant neoplasms, contributing to diagnostic difficulty.

IMTs are characterised by myofibroblastic spindle cell proliferation with variable inflammatory infiltrates [36]. IPTs show plasma cells, lymphocytes, myofibroblasts, and collagen [40]. Dense cellularity, collagen deposition, and fibroinflammatory stroma account for the low T2 signal.

Key differentiating features.

- Unlike malignant tumours, IMTs/IPTs lack destructive invasion and are idiopathic.
- Histology is crucial for diagnosis, as radiological features are nonspecific.
- Clinical context (history of trauma, surgery, or immune-autoimmune triggers) may support IPT over true malignancy [40].

## Granulomatous lesions

### *Granulomatosis with polyangiitis (*Fig. 10*)*

**Fig. 10:**
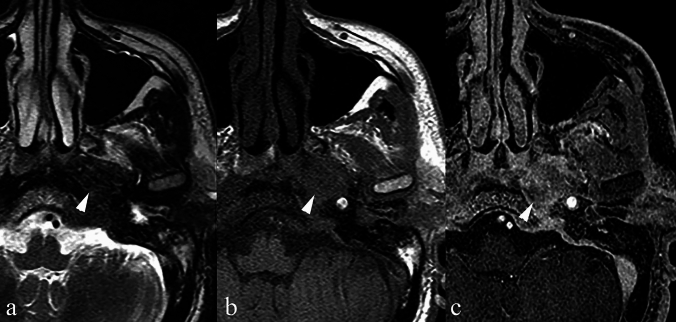
Elderly patient with histologically confirmed granulomatosis with polyangiitis in the left parapharyngeal space. a Axial T2 TSE sequence at the level of the clivus reveals low signal changes in the bone marrow on the left side of the clivus, at its junction with the petrous apex and in the left parapharyngeal space, particularly in the post-styloid compartment (arrowhead). b T1 SE at the same level shows similar changes (arrowhead). c T1 VIBE post-contrast shows modest enhancement of the parapharyngeal space mass (arrowhead)

#### Epidemiology and relevance in the PPS

GPA is a systemic ANCA-associated vasculitis affecting small- and medium-sized vessels. It usually begins in the respiratory tract and may progress to systemic disease [41]. PPS involvement is very uncommon but can occur, as illustrated in Fig. 10.

Imaging features and histopathological correlation.

On MRI, early mucosal inflammatory tissue may appear nonspecific. At later stages, granulomas develop and show T2 hypointensity [43]. Post-contrast imaging often demonstrates modest enhancement of affected tissue.

Histology reveals granulomas with necrosis, vasculitis, lymphocytes, neutrophils, plasma cells, and collagen [42]. The dense cellularity, necrotic components, and fibrosis contribute to low T2 signal intensity [43].

Key differentiating features.

- Initial stages may mimic an acute inflammatory process; progression to T2 hypointense granulomas suggests GPA.
- Systemic clinical features (respiratory tract involvement, renal disease) and positive ANCA serology are essential for differentiation.
- Unlike neoplasms, GPA lesions reflect vasculitis with granulomatous inflammation rather than true tumour growth.

## Pseudolesions

### *Osteomyelitis of the base of the skull (*Figs. 11, 12*)*

**Fig. 11:**
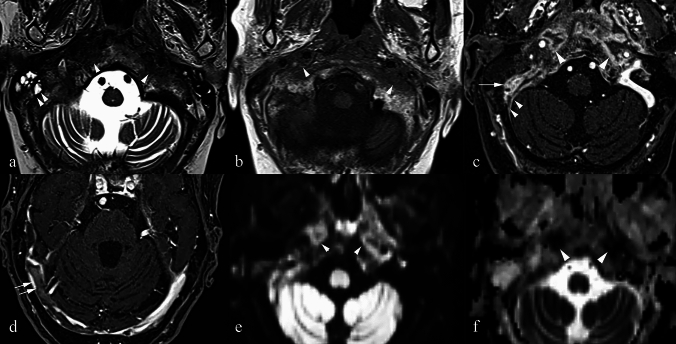
Elderly patient presenting with VII cranial nerve palsy and right-sided otalgia. a Axial T2 TSE at the level of the clivus and petrous apex demonstrates heterogeneous, low signal intensity changes throughout the bone marrow (arrowheads) associated with bilateral mastoiditis worse on the right (double arrowhead). b Axial T1 SE at the same level confirms the pathological marrow replacement c T1 VIBE post-contrast depicts irregular peripheral rim enhancement within the clivus and both petrous apices (single arrowheads) and lack of contrast filling of the right sigmoid sinus in keeping with thrombosis (arrow). Dural thickening and enhancement of the tentorium cerebelli is also appreciable on the ipsilateral side just beneath the thrombosed sigmoid sinus (double arrowhead). d Axial T1 VIBE post-contrast at the level of the transverse sinus reveals an extensive filling defect on the right in keeping with propagation of the thrombus beyond the sigmoid sinus described earlier in (c) (double arrow). e Axial DWI B1000 at the level of the petrous apex shows bilateral high signal which corresponds to low values on the ADC Map (f) confirming restricted diffusion (arrowheads)

**Fig. 12:**
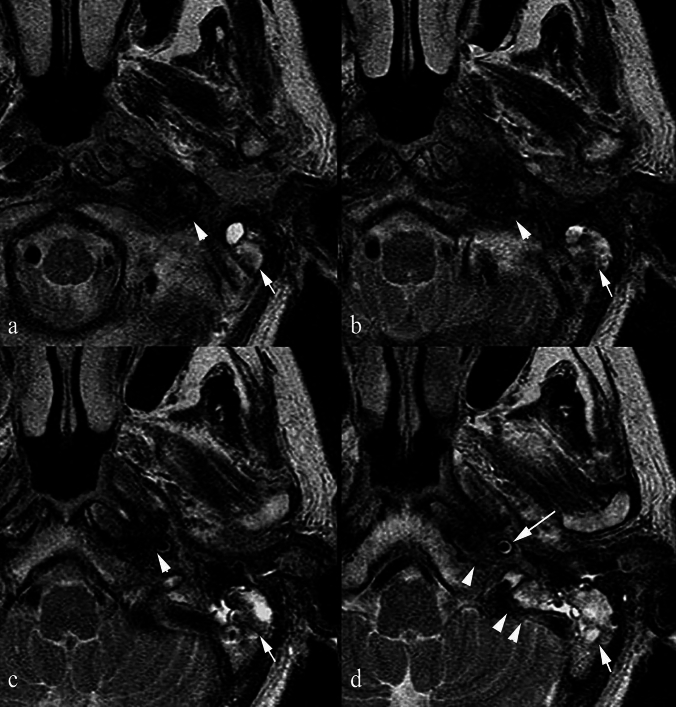
Elderly type II diabetic patient, being treated for malignant otitis externa with antibiotics (confirmed on culture & sensitivity to be due to *Pseudomonas aeruginosa*), presenting with worsening ear and neck pain (a–d) Axial T2 TSE sequences at the level of the nasopharynx and petrous apex demonstrate a left sided effusion in the mastoid air cells (arrow). Hypointense material is observed in the post-styloid compartment (arrowheads) which corresponds to scar tissue secondary to an organising abscess which is encasing the internal carotid artery at the level of the foramen lacerum (arrow in d); abnormal signal within the left sigmoid sinus is present (double arrowhead) raising the suspicion of left sigmoid sinus thrombosis (d). Focal erosions of the clivus along with signal intensity changes of the marrow on the left are also appreciable in (c)

#### Epidemiology and relevance in the PPS

Skull base osteomyelitis (SBO) is an uncommon but serious infection, often complicating otitis externa, oral cavity, pharyngeal, tonsillar, salivary gland, or middle ear infections. It is most frequently seen in older diabetic or immunocompromised patients and is commonly caused by *Pseudomonas aeruginosa* [44]. PPS involvement may occur through spread of infection from adjacent structures.

Imaging features and histopathological correlation.

On MRI, SBO demonstrates T2 hypointensity due to fibrotic and scar tissue, with associated marrow signal changes. Post-contrast images may show irregular peripheral rim enhancement. Diffusion restriction may also be present. CT is useful to assess associated bony erosions.

T2 hypointensity reflects post-inflammatory scarring, fibroinflammatory change, and necrosis within the affected marrow and soft tissues. Chronic osteomyelitis can also result in reduced free water content, contributing to T2 shortening.

Key differentiating features.

- Clinical context (elderly diabetic patients with ear or neck pain, history of otitis externa) is a major clue.
- Unlike malignant tumours, SBO typically shows marrow replacement without a discrete mass.

## Protein Containing Lesions

### Thyroglobulin Containing Lesions

#### Epidemiology and relevance in the PPS

Metastases from papillary and medullary thyroid carcinoma can involve the PPS, though this is rare, with only 112 cases reported in the literature [19]. Recognition is clinically important, as PPS involvement may mimic other entities.

Imaging features and histopathological correlation.

On MRI, thyroglobulin-rich metastases often demonstrate T2 hypointensity. This may coexist with intralesional calcifications and regions of variable T1/T2 signal depending on haemorrhage or colloid content. CT helps confirm calcifications.

Low T2 signal is due to dense proteinaceous colloid material, calcifications, and occasional haemorrhagic byproducts, all of which reduce free water mobility and shorten T2 relaxation times.

Key differentiating features.

- Clinical history of thyroid carcinoma is critical in narrowing the differential.
- The combination of calcifications and proteinaceous material distinguishes these lesions from other hypointense tumours such as melanoma or paraganglioma.
- Unlike benign cystic PPS lesions, these do not show purely fluid signal but instead appear more complex and hypointense on T2.

## Conclusion

**Fig. 13:**
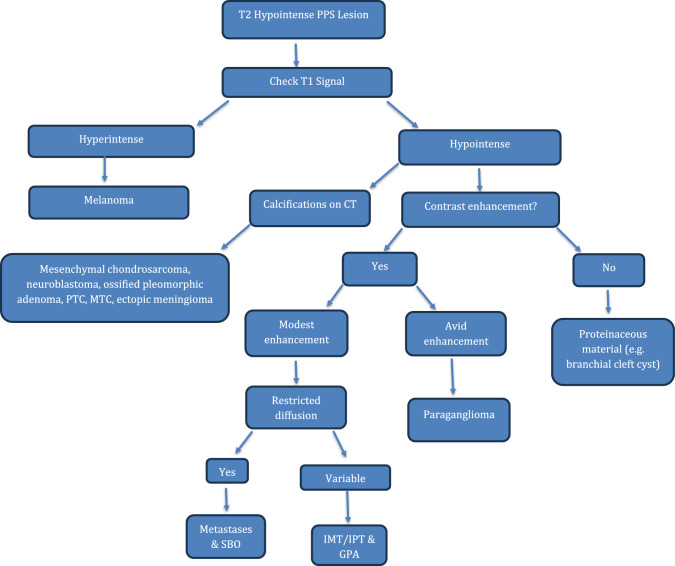
Flowchart highlighting a proposed diagnostic algorithm to aid radiologists when reporting T2 hypointense PPS lesions. *PTC* papillary thyroid carcinoma, *MTC* medullary thyroid carcinoma, *SBO* skull base osteomyelitis, *IMT* idiopathic myofibroblastic tumour, *IPT* inflammatory pseudotumour, *GPA* granulomatosis with polyangiitis

T2 hypointense lesions of the PPS are exceedingly rare. These rare culprits may be melanomas, IMFT, metastases from undifferentiated squamous cell carcinoma and paragangliomas. It is important that the radiologist is aware of these radiological phenotypes. As described, multimodality and multiparametic imaging plays a critical role in narrowing the differential diagnosis (see Fig. 13). Finally, in some case, the imaging features may remain inconclusive, and the pathologist’s role remains crucial in reaching the final diagnosis.

## Abbreviations

*PPS*: Parapharyngeal space
*IMT*: Inflammatory myofibroblastic tumour
*IPT*: Inflammatory pseudotumour
*SB*: Skull base
